# Harnessing oncogenic signaling to enhance oncolytic virotherapy: Lessons from EPAC-encoding vaccinia virus

**DOI:** 10.1016/j.omton.2026.201165

**Published:** 2026-03-10

**Authors:** Stephen Boulton, Siddharth Singh, John C. Bell

**Affiliations:** 1 Ottawa Hospital Research Institute, Ottawa, ON K1H 8L6, Canada; 2 Department of Biochemistry, Microbiology and Immunology, University of Ottawa, Ottawa, ON K1H 8M5, Canada; 3 Department of Medicine, University of Ottawa, Ottawa, ON K1H 8M5, Canada

## Main text

Oncolytic viruses (OVs) exploit intrinsic features of malignant cells to selectively replicate, spread, and induce tumor destruction while stimulating antitumor immunity. A central paradox of this therapeutic modality is that many of the biological programs that enable tumor progression—immune evasion, resistance to apoptosis, metabolic reprogramming, and enhanced migratory capacity—are also highly permissive to viral infection and dissemination.^1^ Rather than representing a liability, this overlap between oncogenic and proviral signaling may be deliberately leveraged to enhance OV performance. In this context, we report the development of an oncolytic vaccinia virus encoding a constitutively active form of exchange protein directly activated by cyclic AMP (cAMP) (EPAC), demonstrating that targeted activation of a tumor-associated signaling axis can markedly improve viral spread, remodel the tumor microenvironment (TME), and synergize with chemotherapy and surgery.^2^

### Pro-tumor signaling as a proviral advantage

**Figure 1:**
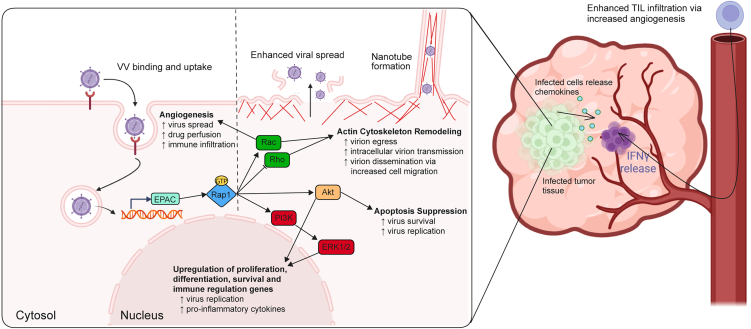
Summary of EPAC signaling pathways involved in oncolytic vaccinia virus therapy

The convergence of cancer-associated signaling pathways and viral fitness has long been appreciated but rarely exploited in a deliberate, mechanistic fashion. Pathways that promote cell survival, proliferation, angiogenesis, and migration are often co-opted by viruses to support entry, replication, and cell-to-cell transmission.^1^^,^^3^ EPAC signaling exemplifies this duality. While EPAC activation has been implicated in cancer progression and metastasis, it also regulates cytoskeletal dynamics, survival, and cellular adhesion—processes that are critical for efficient viral infection (Figure 1).^4^

Previous work demonstrated that pharmacological inhibition of EPAC exerts broad antiviral effects,^5^ whereas systemic EPAC agonism enhances oncolytic virotherapy^6^ but carries risks related to cardiovascular toxicity and potential tumor promotion.^7^ Our study in the *Journal for ImmunoTherapy of Cancer* reframes this apparent contradiction by asking whether EPAC activation can be spatially restricted to tumor sites through viral encoding, thereby capturing its proviral benefits while minimizing systemic risk.^2^ Their findings underscore a broader principle for the field: oncogenic signaling does not merely enable tumor growth but can actively support OV therapeutic efficacy when precisely controlled.

### Encoding tumor-associated signaling to enhance viral spread

A major barrier to effective oncolytic virotherapy remains inefficient intratumoral dissemination, particularly in dense, stromal-rich, or poorly vascularized tumors.^8^ By encoding a constitutively active EPAC construct within vaccinia virus, we demonstrated a substantial enhancement in viral spread both *in vitro* and *in vivo*. Mechanistically, EPAC expression induced pronounced actin cytoskeletal remodeling, formation of intercellular nanotube-like structures, and enhanced syncytia formation—features that facilitate direct cell-to-cell viral transmission.

These effects were especially evident in three-dimensional tumor spheroids and extracellular matrix-rich environments, which better recapitulate the physical constraints of solid tumors than monolayer cultures. Importantly, EPAC expression also improved viral dissemination to distal, non-injected tumors following intratumoral administration, suggesting that local enhancement of migratory and angiogenic programs can translate into systemic virotherapy benefits. Collectively, these data support a strategy in which tumor progression-associated pathways are intentionally harnessed to overcome physical and biological barriers to OV spread.

### Immune remodeling beyond direct oncolysis

While enhanced viral dissemination is a key outcome of EPAC encoding, the therapeutic benefit observed in multiple tumor models cannot be explained by direct oncolysis alone. EPAC-expressing vaccinia virus profoundly reshaped the immune landscape within the TME and systemically. Treatment was associated with increased recruitment of CD8^+^ T cells, alterations in cytokine profiles favoring T cell activation, and enhanced antigen-specific adaptive immune responses.

The link between improved viral spread and immune activation is particularly notable. Increased infection of tumor cells likely augments antigen release and cross-presentation, facilitating systemic immune priming. Moreover, shifts in myeloid populations and cytokine balance suggest a transition toward a more immunostimulatory microenvironment. These findings reinforce the concept that effective OVs function not only as cytolytic agents but also as immune modulators, with tumor-intrinsic signaling pathways playing a critical role in shaping this outcome.

### Synergy with surgery: Immune reconditioning after debulking

One of the most striking findings of the study is the profound synergy between EPAC-encoding vaccinia virus and partial surgical resection in a metastatic melanoma model. Whereas the virus alone conferred limited benefit in this setting, perioperative administration dramatically reduced tumor recurrence, prevented metastatic outgrowth, and significantly improved survival.

This effect likely reflects immunological reprogramming that occurs following tumor debulking. Surgery reduces tumor burden and immunosuppressive pressure while releasing tumor antigens, creating a window during which immune responses can be redirected. EPAC-expressing OV therapy appears to capitalize on this window by enhancing antigen presentation, promoting adaptive immunity, and reducing suppressive myeloid-derived populations that are known to expand after surgical stress.^9^ These observations position OVs as promising perioperative immunotherapies rather than solely end-stage interventions.

### Viral transgenes as localized substitutes for systemic drugs

A particularly important conceptual advance highlighted by this work is the use of viral transgenes to functionally replace systemic pharmacological interventions. Instead of administering small-molecule EPAC agonists—agents associated with off-target effects and potential tumor-promoting activity^7^—we achieved comparable or superior outcomes through localized expression of a constitutively active EPAC construct within infected tumor cells.

This approach affords precise spatial control, confines pathway activation to the tumor, and avoids systemic toxicity. Notably, despite EPAC’s established links to cancer progression, no increase in off-target viral replication, tumor aggressiveness, or toxicity was observed. This finding emphasizes the value of genetic control over signaling pathways that are otherwise difficult to manipulate safely with systemic drugs.

A potential limitation of this strategy is that EPAC activation is restricted to infected cancer cells, potentially limiting the extent of tumor-wide priming compared with systemic agonist administration. However, broader priming would also activate EPAC signaling in healthy tissues, increasing susceptibility to viral infection, and raising safety concerns. The absence of increased toxicity or loss of tumor specificity in this study likely reflects this intentional restriction. This trade-off reframes limited transgene reach not as a shortcoming but as a safety feature that preserves therapeutic index. It also raises important questions for future OV design regarding the optimal balance between tumor priming and systemic risk.

### Implications for next-generation OV design

Taken together, our findings support a paradigm for the next generation of oncolytic virotherapy: one in which tumor-associated signaling pathways are deliberately encoded to enhance viral fitness, remodel the TME, and synergize with standard-of-care treatments such as chemotherapy and surgery. EPAC represents a compelling example of a shared dependency between cancer progression and viral replication that can be therapeutically exploited. More broadly, this work invites systematic exploration of other oncogenic signaling nodes that may similarly enhance OV performance when precisely controlled. As the field continues to integrate virology, immunology, and cancer biology, rational engineering of host signaling pathways within OVs may prove essential for overcoming resistance and unlocking the full therapeutic potential of oncolytic virotherapy.

## Declaration of interests

The authors declare no competing interests.
